# Evolution of Placental Hormones: Implications for Animal Models

**DOI:** 10.3389/fendo.2022.891927

**Published:** 2022-05-25

**Authors:** Anthony M. Carter

**Affiliations:** Cardiovascular and Renal Research, Institute of Molecular Medicine, University of Southern Denmark, Odense, Denmark

**Keywords:** gene duplication, immunology of pregnancy, mammals, physiology of pregnancy, placentation

## Abstract

Human placenta secretes a variety of hormones, some of them in large amounts. Their effects on maternal physiology, including the immune system, are poorly understood. Not one of the protein hormones specific to human placenta occurs outside primates. Instead, laboratory and domesticated species have their own sets of placental hormones. There are nonetheless several examples of convergent evolution. Thus, horse and human have chorionic gonadotrophins with similar functions whilst pregnancy-specific glycoproteins have evolved in primates, rodents, horses, and some bats, perhaps to support invasive placentation. Placental lactogens occur in rodents and ruminants as well as primates though evolved through duplication of different genes and with functions that only partially overlap. There are also placental hormones, such as the pregnancy-associated glycoproteins of ruminants, that have no equivalent in human gestation. This review focusses on the evolution of placental hormones involved in recognition and maintenance of pregnancy, in maternal adaptations to pregnancy and lactation, and in facilitating immune tolerance of the fetal semiallograft. The contention is that knowledge gained from laboratory and domesticated mammals can translate to a better understanding of human placental endocrinology, but only if viewed in an evolutionary context.

## 1 Introduction

The primary function of the placenta is to aid exchange of respiratory gases and nutrients between parent and embryo. In addition, because embryonic and fetal tissues express paternal genes, the placenta fashions molecules that modify maternal immune responses, which otherwise might cause rejection of the semiallograft (1). Finally, the placenta secretes hormones to maintain uterine quiescence, alter maternal metabolism, and influence other aspects of maternal physiology. There is an extensive literature on gas and nutrient exchange and on placental and uterine immunology (2). Less is known, however, about the endocrine functions of the placenta (3). One impediment to research is that many protein hormones specific to placenta are restricted to discrete lineages rather than being widely distributed among mammals.

In this review, the focus is on protein hormones that arose through gene duplications, such as those derived from growth hormone, prolactin and luteinizing hormone. Intriguingly, none of these hormones is widely distributed across mammals although hormones with similar properties have evolved in different lineages. As an example, equine chorionic gonadotrophin is known only from equids, whereas human chorionic gonadotrophin evolved in the lineage of anthropoid primates. Steroid hormones secreted by the placenta have far-reaching effects in mammals but are not a focus of this review. However, placental protein hormones interact with steroids and prostaglandins, sometimes in sequence, as described in later sections.

### 1.1 Placentation

Fetal access to the maternal circulation is dependent on the type of placentation, particularly the interhaemal barrier separating maternal and fetal circulations. In human placenta, this comprises only fetal tissues as the villous trophoblast faces an intervillous space filled with maternal blood. This is one kind of haemochorial placenta. In a more common type, found in many rodents, the maternal blood flows in trophoblast-lined blood channels. In endotheliochorial placentas, trophoblast reaches the maternal capillaries, and placental hormones need to cross the capillary endothelium. Epitheliochorial placentas appear to offer a greater challenge since several layers of fetal and maternal tissue separate the two blood streams. However, as described below, access to maternal tissues can be gained by trophoblast invasion, as in equids, or through fusion of trophoblasts with uterine epithelium, as in ruminants.

The trophoblast and other placental tissues are fetal in origin (4) and express paternal genes. Therefore, trophoblast invasion of the uterine wall challenges the maternal immune system. Since several placental hormones are thought to modulate immune responses, some authors tie their evolution to the degree of invasiveness (5, 6). It should be remembered, however, that the Grosser classification defines the tissue layers of the interhaemal barrier and is not an index of invasiveness (7, 8).

The hormones under discussion are products of the definitive, chorioallantoic placenta. There are other fetal membranes and they vary across mammals (9). In rodents and some other orders, there is a yolk sac placenta that persists to term. It has an epithelium, endodermal in origin, that faces the uterine cavity. This serves mainly for uptake of maternal secretions and antibodies. Whilst the yolk sac does synthesize hormones and hormone-binding proteins, such as transthyretin, these are secreted across the basolateral surface towards the fetal circulation (10).

### 1.2 Mammalian Taxonomy

Nineteen orders of eutherian mammals are currently recognized (11). Based on genomics, they can be assigned to four lineages or superorders (11). Best characterized from the perspective of placental endocrinology is Euarchontoglires, which includes rodents and primates. For Laurasiatheria, a fair amount is known about placental hormones in domesticated species within the orders of even-toed and odd-toed ungulates (Artiodactyla and Perissodactyla). In contrast little is known about placental hormones in bats although Chiroptera is the second most speciose order of mammal (12). The two other superorders are Afrotheria, which includes elephants and tenrecs, and Xenartha, which comprises sloths, anteaters and armadillos. There are few observations on placental hormones in these mammals although some studies have been made on elephants (13). A guide to taxonomic terms used in this review is given in **Table 1**.

**Table 1:** Terminology of eutherian mammals encountered in this review.

Term	Taxonomic level	Remarks and examples
Afrotheria	Superorder	6 orders
Proboscidea	Order	Elephants
Hyracoidea	Order	Hyraxes
Xenarthra	Superorder	2 orders
Euarchontoglires	Superorder	5 orders
Primates	Order	14 families
Strepsirrhini	Suborder	Strepsirrhines: lemurs, lorises, galagos
Haplorhini	Suborder	Haplorhines: tarsiers and anthropoid primates
Simiiformes	Infraorder	Anthropoid primates: New and Old World monkeys, apes
Platyrrhini	Parvorder	New World monkeys
Catarrhini	Parvorder	Old World monkeys, gibbons and great apes (including human)
Rodentia	Order	36 families in 5 suborders
Myomorpha	Suborder	2 superfamilies
Muroidea	Superfamily	6 families including cricetid and murid rodents
Cricetidae	Family	Golden hamster, deer mouse
Muridae	Family	Mouse, rat
Hystricomorpha	Suborder	18 families including guinea pigs
Lagomorpha	Order	3 families including rabbits
Laurasiatheria	Superorder	6 orders
Chiroptera	Order	21 families
Yinchiroptera	Suborder	Megabats and 6 other families
Yangchiroptera	Suborder	15 families including vesper bats
Perissodactyla	Order	3 families of odd-toed ungulates including tapir, rhinoceros and equids
Equidae	Family	Horse, zebra
Artiodactyla	Order	24 families of even-toed ungulates (includes whales) including pig, hippopotamus, llama and ruminants
Ruminantia	Suborder	Mouse deer and pecoran ruminants
Pecora	Infraorder	Cattle, water buffalo, sheep, goat, deer, giraffe, pronghorn, wildebeest

For context the number of orders in each superorder is given as well as the number of families in selected orders (11, 12).

## 2 Recognition and Maintenance of Pregnancy

**Figure 1:**
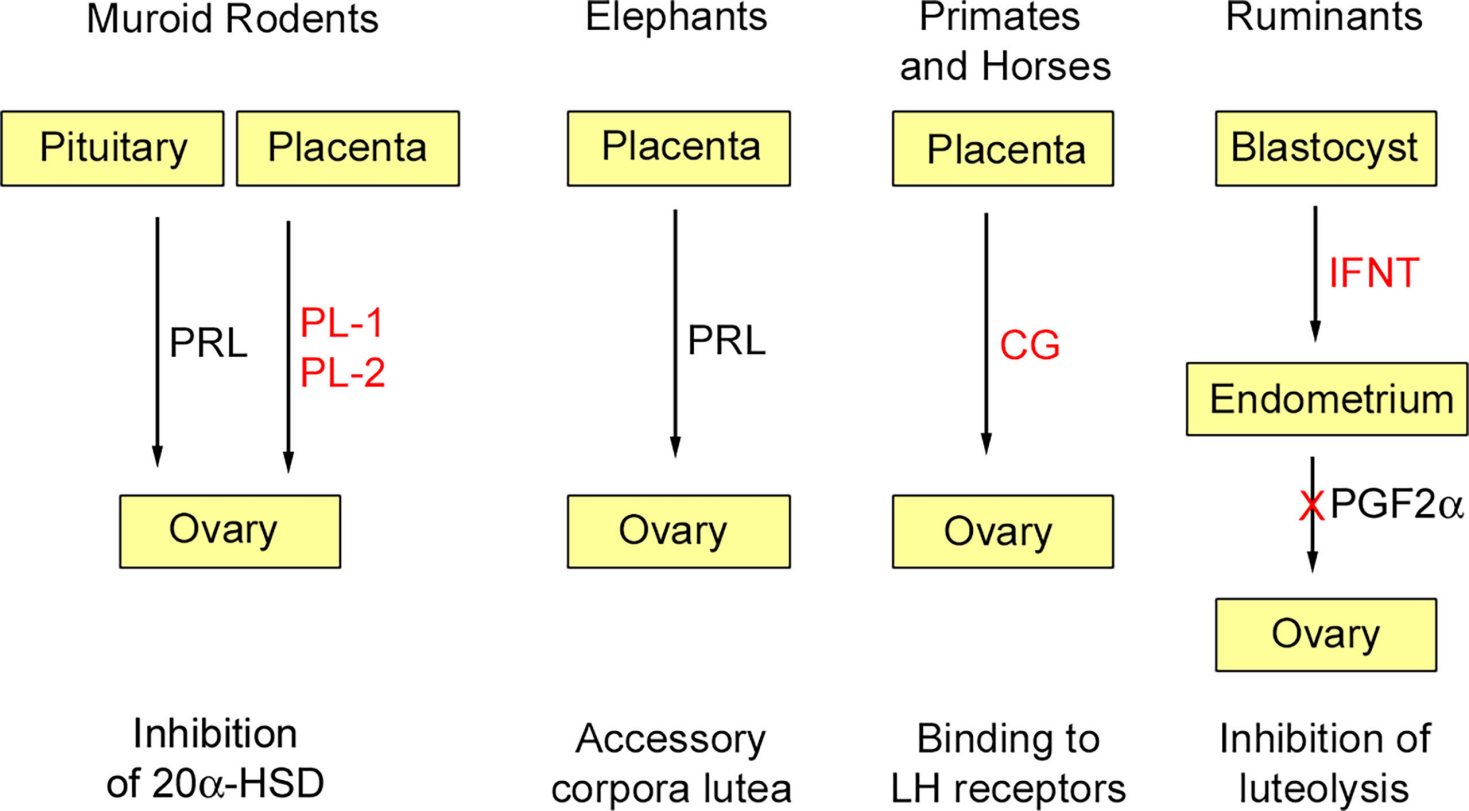
Some luteotrophic factors. Those derived through gene duplication are shown in red. In murid and cricetid rodents (e.g., mouse, golden hamster) pituitary prolactin (PRL) is released in response to coitus and inhibits 20α-hydroxysteroid dehydrogenase (20α-HSD); subsequently this function is assumed by placental lactogens (PL-1, PL-2). In elephants, placental expression of *PRL* is responsible for pregnancy maintenance by accessory corpora lutea. In anthropoid primates and horses, chorionic gonadotropins (CG), derived through duplication of the luteinizing hormone β-subunit, are expressed by trophoblast and maintain luteal function in the early months of gestation. In ruminants, interferon-tau (IFNT) derived by duplication of the *INFW* gene is secreted by the blastocyst and acts on the endometrium to inhibit the luteolytic signal prostaglandin F_2α_ (PGF2α). Reproduced from Physiological Reviews (2) Copyright ^©^ 2012, The American Physiological Society.

At least initially, pregnancy maintenance is contingent upon continued secretion of progesterone from the corpus luteum, and this requires maternal recognition of pregnancy. As long realized, there is no mechanism common to all species (14). In most mammals studied, pregnancy recognition depends on inhibition of a luteolytic factor, prostaglandin F_2α_ (PGF_2α_), secreted by the uterus. Thus, maternal recognition of pregnancy usually depends on inhibition of PGF_2α_ secretion; this is achieved by cytokines or hormones secreted by the blastocyst or placenta. Experiments in guinea pigs (*Cavia porcellus*) showed that luteolysis was inhibited in the ovary ipsilateral to a pregnant horn, but not on the same side as a sterile horn [e.g. (15)]. A close apposition of ovarian arteries to the utero-ovarian vein was then shown for several species, including guinea pig and sheep (*Ovis aries*) (16, 17). Further work, summarized in a recent review (18), showed how transfer of PGF_2α_ from vein to artery is aided by the counter current arrangement of these blood vessels. This route is not available to all mammals. In the mare, for example, PGF_2α_ reaches the ovaries by the systemic route. In human and some other primates, the source of PGF_2α_ is intra-ovarian rather than uterine and its synthesis is decreased in the presence of chorionic gonadotrophin (19). Several of the luteotrophic factors discussed below are shown schematically in **Figure 1**.

### 2.1 Pecoran Ruminants

In ruminants, the signal for pregnancy recognition is interferon tau, which is secreted by the trophectoderm of the elongated blastocyst during its long sojourn in the uterine lumen. The *IFNT* gene arose in the lineage of pecoran ruminants through duplication of the *IFNW* gene (20). In the process, *IFNT* lost the viral control elements of the promotor region and acquired sequences responsible for trophectoderm-specific expression (21, 22). Interferon tau binds to receptors in the endometrium and acts to suppress secretion of PGF_2α_ thus preventing luteolysis (23, 24). It is also detectable in uterine venous blood and may act on extrauterine tissues (25). Recent work has focussed on the direct effects of interferon tau on the transcriptome of the corpus luteum. Both the luteinized large cells and the luteal endothelial cells respond to interferon tau and the net effect is downregulation of luteolytic factors, promotion of cell survival and vascular stability (26). *IFNT* is absent in non-ruminant species of Artiodactyla, such as pig (*Sus scrofa*), hippopotamus (*Hippopotamus amphibius*) and llama (*Lama glama*) (27). The pregnancy recognition signal in pigs is estrogen, primarily estradiol-17β, secreted by the trophectoderm of the filamentous blastocyst (28).

### 2.2 Equids

In the horse (*Equus caballus*), the developing blastocyst is enclosed in a glycoprotein capsule that moves about the uterus. Blastocyst motility is essential to maintenance of pregnancy. A hitherto unidentified factor inhibits release of PGF_2α_ from the uterus (29). A recent overview concluded that maternal recognition of pregnancy in the horse involves a combination of chemical and mechanical signalling through multiple pathways (30). After implantation, between days 36 and 38 of gestation, trophoblast migrates from the chorionic girdle of the developing placenta into the endometrium and there forms the endometrial cups, which secrete equine chorionic gonadotrophin (eCG) (31, 32). Unlike in primates, a single gene codes for the β-subunit of pituitary LH and placental CG. It evolved in the equid lineage through acquisition of an extended carboxy-terminal peptide that is key to placental expression (33, 34). Placental expression occurs in the donkey (*Equus asinus*) and Plains zebra (*Equus quagga*) (34, 35), but not in other perissodactyls such as the Central American tapir (*Tapirus bairdii*) and various species of rhinoceros (36). Together with pituitary FSH, eCG stimulates development of accessory corpora lutea, which support pregnancy until about day 70, after which pregnancy is maintained through placental secretion of progestins such as dihydroprogesterone (DHP). For a fuller consideration of the endocrinology of pregnancy in the mare, including estrogen production by the fetoplacental unit, the reader is referred to an excellent review by Conley (37).

### 2.3 Anthropoid Primates

There is a superficial resemblance between horse and human in that pregnancy maintenance depends initially on hCG, which is secreted by the syncytiotrophoblast, and then on placental secretion of progesterone. Although hCG rescues steroid synthesis by the corpus luteum (38), its role may be short-lived (39). There is no chorionic gonadotrophin in lemurs, lorises or tarsiers. Duplication of the pituitary LH β-subunit gene, which presaged evolution of chorionic gonadotrophin, occurred in the lineage of anthropoid primates (40). Several copies of the CG β-subunit gene are found in anthropoid primates, but many lack placental expression (41). In human gestation, the plasma concentration of hCG rises rapidly 4 weeks after implantation and peaks at 8 to 10 weeks (42). Subsequently, pregnancy is maintained through placental secretion of progesterone. An enduring hypothesis is that human parturition is triggered by progesterone withdrawal (43). The current view favours a functional progesterone withdrawal resulting from an increase in the ratio between the two progesterone receptors (PR-A/PR-B ratio) and mediated by prostaglandins, although the full picture is considerably more complex (44, 45).

It should be remarked that hCG has actions other than maintenance of the corpus luteum (3). For example, hCG induces proliferation of the uterine natural killer cells that play a key role in maternal-fetal interactions within the placental bed (46).

### 2.4 Murid and Cricetid Rodents

In mouse (*Mus musculus*), rat (*Rattus norvegicus*), and golden hamster (*Mesocricetus auratus*), rescue of the corpus luteum is through pituitary secretion of prolactin (PRL), which requires stimulation of the uterine cervix during coitus. As pregnancy proceeds, PRL is supplemented by placental lactogens (PLs). PRL, PL-I and PL-II act by silencing expression of 20α-hydroxysteroid dehydrogenase, which otherwise would catabolize progesterone (47). The trophoblast giant cells secrete PL-I in mid-gestation and PL-II during the last half of gestation (48). In rodents, placental lactogens arose through duplication of the *Prl* gene and in mouse they are encoded by *Prl3d1* (PL-I) and *Prl3b1* (PL-II). A variant form of PL-I in the rat is encoded by *Prl3d4*. Further functions of PLs and prolactin-like proteins in rat and mouse are discussed in a later section.

Evolution of placental lactogens in rodents has yet to be explored in depth. *Pl1*, *Pl2*, and variants thereof, have been documented in cricetid rodents: the golden hamster (49) and two species of deer mouse (*Peromyscus maniculatus* and *P. polionotus*) (50). Thus, based on current phylogeny, *Pl1* and *Pl2* must have been present in the common ancestor of Muridae and Cricetidae, which together account for 94% of muroid diversity (51). Without further data, it is not possible to pinpoint when and where placental lactogens emerged in this lineage of rodents.

### 2.5 Guinea Pig

The guinea pig and other hystricomorph rodents have a long gestation and give birth to well-developed young. They differ in this respect from mouse and rat. Maintenance of the corpora lutea in the first weeks of guinea pig pregnancy has been ascribed to a chorionic gonadotrophin [evidence summarized in (52)]. In addition, spongiotrophoblast from the interlobular areas of the placenta secretes prolactin-like proteins (53). The ovaries are not required after day 21 of gestation when pregnancy maintenance depends on placental secretion of progesterone (54). As in women, there is no decline in plasma progesterone prior to birth suggesting guinea pig as a promising model for research on parturition (44).

### 2.6 Elephant

The prolactin gene went through a period of rapid evolution in the African savannah elephant (*Loxodonta africana*) and rock hyrax (*Procavia capensis*), but without gene duplication (55). Nonetheless, placental expression of *PRL* has been suggested for both African and Asian elephants (*Elephas maximus*) based on immunostaining with an antibody raised against human prolactin (56, 57). Gestation in elephants is maintained by large accessory corpora lutea and the luteotrophic factor may well be PRL derived from the placenta (58).

## 3 Maternal Adaptations for Pregnancy and Lactation

Placental hormones have diverse yet poorly understood effects on maternal physiology (3). They stimulate growth of the uterine glands and secretion of histotroph (uterine milk), which is an important source of fetal nutrition, especially in species with epitheliochorial placentation (59). They also have far-reaching effects on maternal metabolism that ensure an adequate supply of nutrients to the fetus. Placental lactogens are among a plethora of hormones that support differentiation of the mammary glands preparatory to lactation (60). There are also behavioural effects such as nest-building in rabbits (61).

The hormones responsible for these actions include placental lactogens. They occur in primates, rodents and ruminants but are the result of separate evolutionary trajectories and are derived from different genes. Therefore, they cannot be assumed to have identical functions. Known effects of PRL-related hormones on placental development are considered here though those actions may be paracrine rather than endocrine Also considered in this section are placensin, recently described as a human placental hormone, and the pregnancy-associated glycoproteins of artiodactyls.

### 3.1 Primates

Duplication and placental expression of the growth hormone gene is a distinctive feature of anthropoid primates. A cluster of five genes is found on human chromosome 17. One codes for pituitary growth hormone (*hGH-N*), another for placental growth hormone variant (*hGH-V*) and two for placental lactogens, also known as chorionic somatomammotropic hormones (*CSH1*/*hCS-A*, *CSH2*/*hCS-B*) (62). The appellation placental lactogen is supported by their much greater affinity for the PRL receptor than the GH receptor (63).

#### 3.1.1 Placental Growth Hormone

Placental GH is secreted from 24-25 weeks of gestation, reaches a plateau at 34-35 weeks, and is maintained to term. It suppresses secretion of pituitary GH from 24-25 weeks (64). Secretion of placental GH is continuous (65) whereas pituitary GH is secreted in pulses. Since GH promotes gluconeogenesis, lipolysis and anabolism, it is though that the placental variant increases nutrient availability to the placenta and fetus (66, 67). Thus, maternal insulin resistance develops during mid- to late human pregnancy in response to placental GH, thereby ensuring availability of maternal glucose for placental transfer [reviewed in (67)]. Placental growth hormone is not secreted to the fetal circulation (68).

Many actions of GH are mediated through *STAT5B*, which upregulates transcription of insulin-like growth factor 1 (*IGF1*) (69). In addition, the syncytiotrophoblast of human placenta expresses *IGF2* (70) and the maternal plasma concentration of IGF-2 rises throughout pregnancy. The actions of IGFs are mediated through their receptors *IGF1R* and *M6P/IGF2R* as well as through the insulin receptor. Several IGF binding proteins regulate their availability. For closer consideration the reader is referred to appropriate reviews (67, 70–72).

#### 3.1.2 Placental Lactogens

Human PL is found in maternal plasma at around 6 weeks and reaches a plateau by 32-35 weeks of gestation (68). Towards term, the secretion rate of hPL is about 1 g/day, exceeding that of any other peptide hormone (73). It binds preferentially to the PRL receptor (63). However, although levels of hPL greatly exceed those of PRL, a role in the secretory differentiation of the human mammary gland has yet to be determined (60). Thus, the increase in urinary lactose, reflecting the capacity of the breast to make lactose, correlates with PRL levels but not hPL levels (74). Pregnancy proceeds to term even in the absence of circulating hPL, although fetal outcomes vary (68). In humans most placental hormones are secreted by the maternal-facing syncytiotrophoblast: secretion is unidirectional. Human PL is an exception and is found in the fetal circulation (75).

It may be noted that *Prl* itself is expressed in the uterine decidua of anthropoid primates including brown-headed spider monkey (*Ateles fusciceps*), rhesus macaque, and human (76).

#### 3.1.3 Growth Hormone Locus in Nonhuman Primates

Most mammals have a single growth hormone gene expressed in the pituitary. This is also true of strepsirrhine primates such as the slow loris (*Nycticebus pygmaeus*) (77) and tarsiers (*Carlito syrichta* and *Cephalopacus bancanus*) (78). Phylogenetic analysis infers there was a single gene in the common ancestor of New World and Old World monkeys, although it may already have attained placental expression (79). Gene duplication occurred separately in the two lineages (79). Multiple *GH* genes are found in New World monkeys, for example 8 genes and pseudogenes in the common marmoset (*Callithrix jacchus*) (80), which is an important animal model (81). At least three genes are expressed in the placenta of the brown-headed spider monkey (*Ateles fusciceps*) (79). Among Old World monkeys, baboon placenta expresses placental growth hormone (*GH-2*) and two PLs (*CSH*s) (82) and rhesus macaque (*Macaca mulatta*) has a cluster of six *GH*-like genes, four of which are expressed in the placenta (83). Like human, chimpanzee (*Pan troglodytes*) and lowland gorilla (*Gorilla gorilla*) have two *GH* genes and 3-4 *CSH*-like genes (84, 85).

#### 3.1.4 Placensin

The *FBN1* gene encodes a structural protein, fibrillin-1, and a secreted protein, asprosin. Its paralogue *FBN2* was recently shown to be highly expressed by cyto- and syncytiotrophoblast of human placenta and given the name placensin (86). Based on its ability to stimulate glucose secretion and gluconeogenesis in primary hepatocytes, it was suggested that placensin plays a role in metabolic homeostasis during pregnancy. It should, however, be noted that the gene is expressed at very low levels in mouse placenta and *FBN2* is highly conserved across vertebrates (86).

### 3.2 Rodents

As already noted, PRL, PL-1 and PL-2 maintain corpus luteum function, which is a prerequisite for mammary development. In addition, they act through the PRL receptor (coded by *Prlr*) to promote lobuloalveolar growth, differentiation, and milk protein gene expression (87, 88). Until quite recently, the metabolic effects of rodent PLs were unclear. However, a comparison of mouse mutants lacking either *Prl* or *Prlr* indicated that PLs rather than prolactin were essential for maintenance of adequate glucose levels during gestation (89).

A large cluster of *PRL*-like genes occurs in rat and mouse (90, 91). Many of these are orphan ligands or act through non-classical pathways rather than through the PRL receptor (92). They include genes coding for proliferin (*Prl2c2*) and proliferin-related protein (*Prl7d1*). Proliferin promotes angiogenesis and is expressed by the trophoblast giant cells during development of the placental labyrinth (93). In contrast, proliferin-related protein, which is expressed by cytotrophoblasts of the junctional zone, is anti-angiogenic. At midgestation there is decrease in proliferin and increase in proliferin-related protein that may restrict further vascularization (93). Whilst these actions are paracrine, both proteins are secreted to the maternal circulation.

Several of the PRL-like proteins (PRL-A, -B, and -C) are expressed by the trophoblasts that invade the mesometrial triangle in the last week of gestation. This coincides with the disappearance of uterine natural killer (uNK) cells and PLP-A has been shown to bind to uNK cells and suppress their synthesis of interferon-gamma (94).

Rodents do not have a placental growth hormone. However, in the mouse, pituitary secretion of GH rises at mid-gestation coincident with development of the chorioallantoic placenta (95). The increase has been attributed to a placental factor. Subsequent work ruled out placentally derived acyl-ghrelin (96), but placental secretion of GH- releasing hormone (GHRH) remains a possibility (95). Finally, it is noteworthy that *Prl* is expressed by uterine decidua in murid rodents (76), as in anthropoid primates, although this is another incidence of convergent evolution (76).

### 3.3 Artiodactyls

Most research on placental hormones of artiodactyls has focussed on domesticated species such as cattle (*Bos taurus*), sheep, and goat (*Capra hircus*). These and other ruminants, including the basal tragulids or mouse deer (*Tragulus javanicus* and *T. napu*) (97), feature binucleate trophoblast cells (BNCs) that synthesize prolactins and pregnancy-associated glycoproteins (PAGs). As first shown in cattle (98), BNCs can fuse with uterine epithelial cells to form a short-lived trinucleate cell that delivers the hormones to maternal tissues. Trinucleate cells have been demonstrated in species from three further families, including in white-tailed deer (*Odocoileus virginianus*), Northern giraffe (*Giraffa camelopardalis*) and pronghorn (*Antilocapra americana*) (99, 100). In sheep, goat, and blue wildebeest (*Connochaetes taurinus*), there is a syncytium that Wooding considers to be a hybrid tissue maintained by continual fusion with BNCs (100, 101). This view has been challenged recently, and it is suggested that uterine epithelial cells undergo apoptosis so that the syncytial layer is entirely trophoblastic in origin (102). If that is the case, the placenta is syndesmochorial according to the Grosser classification (7).

#### 3.3.1 Placental Lactogens

Ovine placental lactogen (oPL) stimulates hyperplasia of the uterine glands and secretion of histotroph (103). There is also evidence that it promotes mammary growth in ewes (104), as does bovine placental lactogen (bPL) in heifers (105). However, oPL affected neither growth nor milk production when given to lactating ewes (106). Additionally, these hormones may be important for fetal growth as oPL is secreted to the fetal circulation in sheep. Indeed, RNA interference studies in sheep support the view that oPL stimulates fetal growth by enhancing transcription of *IGF1* and *IGF2* as well as some IGF-binding proteins (107).

The placental lactogens of ruminants arose through duplication of the *PRL* gene with subsequent expansion of the gene locus. Thus, in cattle there are 8 *PRL*-like genes. One codes for bPL; the remainder for prolactin-related proteins (PRPs) (108, 109). There is considerable sequence divergence between bPL and oPL, and bPL is glycosylated whereas oPL is not (110). Apart from goat and water buffalo (*Bubalus bubalis*), other species have not been investigated at the molecular level. Therefore, it is not possible to determine when ruminant PLs evolved. However, prolactin-like activity has been found in placentas of other ruminants including Northern giraffe and six species of deer (Cervidae) as well as in llama, though not domestic pig (111). In addition, placental lactogens have been demonstrated in BNCs from several species including mouse deer (97), deer (112) and giraffe (113) by immunostaining with antibodies raised against oPL and bPL. Giraffe BNCs stain for PRL itself (113).

#### 3.3.2 Placental Growth Hormone

Placental expression of growth hormone is found in sheep and goat, but is restricted to the caprine lineage (114). Sheep are polymorphic for the gene duplication (115). One allele carries a single gene (*GH1*) and the other has two copies of the duplicated gene (*GH2-N* and *GH2-Z*). Only the latter are expressed in the placenta (116), so individuals that are homozygous for *GH1* lack placental expression. The product of *GH2-Z* has a higher affinity for the GH receptor (115) and oGH has been shown to promote endometrial gland proliferation (117). This suggests that the duplicate gene may confer an advantage on fetuses that carry it. The GH gene locus has a similar structure in the domestic goat (114), but little work has been done on this species.

#### 3.3.3 Pregnancy-Associated Glycoproteins

Most mammals have a single PAG-like gene belonging to the aspartic peptidase family. There have been two rounds of duplication in artiodactyls (118, 119). Products of the first round, referred to as “ancient PAGs,” retain the active site. In cattle, they are expressed mainly by trophoblasts of the intercotyledonary chorion (120). In both pig and cow, the proteins occur at the microvillous junction between uterine epithelium and trophoblast suggesting they may function as linking molecules and be important for fetal-maternal anchorage (121). The second round of gene duplication occurred in ruminants (including mouse deer). In cattle, these “modern PAGs,” are expressed predominantly by BNCs in the placental cotyledons (120). There has been expansion within both clusters with cattle having 21 PAG genes and 20 PAG-like pseudogenes (120). There are comprehensive studies of gene expression in cattle comprising both modern and ancient *PAG*s (120, 122). Comparative studies have been restricted to protein expression using antibodies raised against ovine PAG-1 and bovine PAG-2 (100). PAGs have been included here as several are released from BNCs. It has been speculated that they are involved in immune tolerance (123). Another possible role is pregnancy maintenance (122).

## 4 Immunosuppression

The immunological paradox of pregnancy, to which Medawar drew attention (1), has baffled scientists for nearly seventy years (124, 125). Many placental cytokines and hormones are suggested to contribute to immune tolerance. Here I have chosen to highlight the pregnancy-specific glycoproteins (PSGs) and galectins.

PSGs belong to the carcinoembryonic gene family, which in turn is part of the immunoglobulin gene superfamily. There are two major branches coding, respectively, for cell adhesion molecules (*CEACAM*s) and PSGs (*CEAPSG*s). The function of this large group of proteins is not entirely clear, but PSGs are secreted by trophoblast and are putative immunomodulatory agents. Developments in the last few years have widened the spectrum of PSGs from primates and rodents to horses and bats (5).

Galectins are an ancient group of proteins with a wide variety of functions that in mammals include regulation of immune tolerance at the maternal-fetal interface (126–128). Galectins are predominantly localized to the cytoplasm but are included here because there are placenta-specific galectins in primates, one of which is secreted to the maternal circulation. For a broader consideration of the role of galectins in the female reproductive tract the reader is referred to recent comprehensive reviews (127, 128).

### 4.1 Anthropoid Primates

#### 4.1.1 Pregnancy-Specific Glycoproteins

Human placenta secretes large amounts of PSG (previously named β_1_-glycoprotein). There is a cluster of 10 *PSG* genes in human and similar numbers in great apes and Old World monkeys though only 1-7 in New World monkeys (5). *PSG* genes are absent in lemurs, lorises and tarsiers, so the origin and expansion of *CEAPSG* genes occurred in the lineage of anthropoid primates (5). The biological role of PSGs has not been fully resolved. However, all human PSGs activate transforming growth factor β1 (TGF-β1) in immune cells and may thereby contribute to immune tolerance and vascular remodelling [reviewed in (129)]. PSGs also stimulate proliferation of CD4^+^, Fox3^+^ regulatory T-cells, which is TGFβ1-dependent, further supporting a role in maternal tolerance of pregnancy (46, 130). Human PSGs have a highly conserved RGD peptide motif and bind to integrin α5β1. Since both PSGs and integrin α5β1 are expressed by extravillous trophoblasts, it has been suggested that PSGs can promote trophoblast invasion of the uterine decidua (131).

#### 4.1.2 Galectins

Seven galectins are expressed by the trophoblast of human placenta (132). Of particular interest is a cluster of genes on chromosome 19 unique to anthropoid primates and absent in tarsier, greater galago (*Otolemur garnetti*) and grey mouse lemur (*Microcebus murinus*) (133). There is variation between species. However, particular interest attaches to *LGALS13* found in catarrhines (Old World monkeys and apes), because the gene product (galectin-13 or PP13) is secreted from the villus syncytiotrophoblast to the intervillous space and reaches the uterine decidua. In first trimester human pregnancies, aggregates of galectin-13 are found in necrotic zones close to the decidual veins. These appear to attract, activate, and induce apoptosis of maternal immune cells that might otherwise attack invading trophoblast (134). Intriguingly, PSG-1 is a ligand for galectin-1 (135).

### 4.2 Murid Rodents

Although rodent and primate PSGs are thought to have evolved from a *CEACAM-1*-like gene, the two gene families arose independently through convergent evolution. Orthologous genes are found only within rodents (mouse and rat) or primates (human and baboon) (136). The *Psg* locus of the mouse comprises 17 genes and has been explored in detail (137). The rat *Psg* locus has evolved less rapidly and includes eight genes (136). Trophoblast giant cells express *Psg22* in the first half of mouse pregnancy, whereas the spongiotrophoblast expresses *Psg16*, *Psg21* and *Psg23* in the second half (138). Mouse Psg23 can activate latent TGFβ1 (139) and likely increases the availability of regulatory T-cells, as shown by administering recombinant human PSG1 to mice (140).

PSGs do not occur in the guinea pig (6), but have not been sought in other rodents so it is not known if they evolved in the murid lineage or a deeper branch.

### 4.3 Equids

In the horse, PSGs evolved through expansion from a *CEACAM1*-like ancestral gene to a cluster of some 17 genes coding for secreted proteins (141, 142). Five are known to be expressed in the trophoblast of the endometrial cups (142). As mentioned above, the cups are formed by invasive trophoblast and are responsible for secretion of eCG. The endometrial stroma surrounding the cups is heavily infiltrated by CD4+ and CD8+ T-cells as well as by macrophages and natural killer cells (143, 144), yet the cups survive until at least 100 days of gestation. Immunosuppression by secreted PSGs is a plausible hypothesis (142). It is thought that regulatory T-cells play a role in tolerance of the invasive trophoblast (145). Since at least one of the equine PSGs can activate TGFβ1, they may contribute to differentiation of regulatory T-cells (146) as shown for human and mouse (130). PSG expression has not been examined beyond 36 days (142) and it would be interesting to know if the putative protection is withdrawn at a later stage when the cups become necrotic and eventually are sloughed off. It is not known if PSGs occur in other perissodactyls, such as tapirs and rhinoceroses.

### 4.4 Bats

Chiroptera is the most speciose order after rodents. Based on molecular and morphological evidence it can be divided into two clades: Yinpterochiroptera includes megabats and six families of echo-locating bats; the remaining orders constitute Yangochiroptera (147, 148). Expansion of *CEACAM* genes has occurred in Yinpterochiroptera, but *CEAPSG*-like genes have been found only in three families of Yangochiroptera (149). One cluster of putative *PSG* genes occurs in the Natal long-fingered bat (*Miniopterus natalensis*), the common moustached bat (*Pteronotus parnellii*) and four species of vesper bat. A second cluster is restricted to the vesper bats. It must be stressed that bat PSGs were identified from genomic data. Further evaluation will require demonstration of placental expression of these genes.

## 5 Discussion

The most remarkable thing about placental peptide and protein hormones is that each is restricted to a rather narrow group of mammals. Only ancient PAGs occur throughout an entire order (**Table 2**). Apparent similarities, as between humans and rodents, are the result of convergent evolution. Thus, placental lactogens were derived either from growth hormone or prolactin. They have some properties in common but significant differences that may be related to pregnancy duration. Before considering the implications for animal models, we shall consider gene duplications and the subsequent expansion to multigene families.

**Table 2:** Protein hormones secreted by the placenta that evolved through gene duplication.

Hormone	Derivation	Distribution
Interferon-τ (*IFNT*)	Interferon-ω (*IFNW*)	Pecoran ruminants (Infraorder)
Placental lactogens (*hCS-A* and *hCS-B* in human)	Growth hormone (*GH*)	Anthropoid primates (Infraorder) with separate trajectories in New World monkeys (Parvorder) and Old World monkeys plus apes (Parvorder)
Placental growth hormone (*GH2-Z*)	Growth hormone (*GH2-N*)	Sheep and goat (Family)
Placental lactogens (e.g., PL-I (*Prl3d1*) and PL-2 (*Prl3b1*) in mouse and rat)	Prolactin (*PRL*)	Murid and cricetid rodents (Families with common root)
Placental lactogens (e.g. *CSH2* in cattle) and prolactin-like proteins	Prolactin (*PRL*)	Ruminants (Suborder)
Chorionic gonadotropin β-subunit (e.g., *CGB1* in human)	LH β-subunit (*LHB*)	Anthropoid primates (Infraorder)
Chorionic gonadotropin β-subunit (*eCGβ* in horse)	LH β-subunit (*LHB*)	Equids (Family); evolution without gene duplication
Pregnancy-specific glycoproteins (*CEAPSG*s)	*CEACAM-1*-like gene	Anthropoid primates (Infraorder)
Pregnancy-specific glycoproteins (*CEAPSG*s)	*CEACAM-1*-like gene	Murid rodents (Family)
Pregnancy-specific glycoproteins (*CEAPSG*s)	*CEACAM-1*-like gene	Equids (Family)
Pregnancy-specific glycoproteins (*CEAPSG*s)	*CEACAM-1*-like gene	Bats (Some families of Suborder Yangochiroptera)
Pregnancy-associated glycoproteins (*PAG*s)	An aspartic proteinase	Artiodactyls (Order) but “new PAGS” confined to ruminants (Suborder)

### 5.1 Gene Duplication

Placental protein hormones evolved through duplication of existing genes including those coding for pituitary hormones (**Table 2**). Gene duplication often is preceded by a burst of rapid change in nucleotide sequence (87, 150). These bursts can be interpreted as reflecting adaptation of the gene product for a new function, alternating with reversal towards the original function. Repeated cycles of change would lead to accumulation of substantial changes. The process would end once gene duplication allowed a second protein to adopt the new function (87).

### 5.2 Multigene Families

After initial duplication, the genes of many placental hormones have undergone further expansion to create multigene families. This is a contrast to adult hormones, which usually are encoded by a single gene (6). One explanation has been formulated in terms of parent-offspring conflict; a hypothesis based on conflicting priorities for allocation of maternal resources to the offspring. Paternal genes evolve to promote nutrient supply and improve survival of the neonates, while maternal genes evolve to conserve maternal resources for subsequent pregnancies. These ideas were developed to explain the evolution of placental lactogens and their receptors (151). Parent-offspring conflict has also been suggested as a plausible explanation for the expansion of PSG genes in human (eleven genes), mouse (seventeen), and horse (seven genes) (6). It has also been alluded to with respect to the multigene family of PAGs (119).

An alternative hypothesis suggests that bursts of rapid evolution in genes and their repeated duplication reflect the arms race between pathogens and their mammalian hosts. After decades of research on the evolution of growth hormone, prolactin and their receptors, Wallis concluded this to be a plausible explanation for his findings (150). He hypothesized that viruses could gain access to cells by binding to these hormones and then be internalized with the hormone-receptor complex. Thus, the hormones would evolve rapidly to hinder binding of the virus. Gene expansion, as seen with placental lactogens and their kin, would result in multiple species that could act as viral decoys. Human PL reaches very high levels in the second half of gestation, 100 times greater than GH in nonpregnant humans, yet its physiological significance remains unclear. However, a high concentration of hPL would decrease the odds of a virus fastening to a molecule that was to be internalized after receptor binding. The downside to this hypothesis is that viruses capable of binding to growth hormone or prolactin have yet to be identified (150, 152).

Here it is worth noting that CEACAMs are thought to act as decoy receptors and PSGs may act in a similar fashion, so this is an alternative explanation for expansion of PSG gene loci (149). However, as with the PL locus, there is no clear evidence that PSGs bind to microorganisms (6).

### 5.3 Genomic Imprinting

Fetal growth is regulated by the insulin-like growth factor system with IGF2 as a key factor (67). *IGF2* is expressed in the adults of most amniotes (reptiles, birds and mammals), including human (153), whereas in mouse and rat *Igf2* is expressed in placenta but is largely absent in adults. Both *IGF2* and the *M6P/IGF2R* receptor are imprinted genes in most mammals including marsupials (154). In mice the phenotype of the conceptus, including the trophoblast, is determined by the paternal allele of *Igf2* and the maternal allele of *Igf2r*. This reciprocal imprinting has been interpreted in terms of parent-offspring conflict: the kinship theory of genomic imprinting (155). It has limited application to human pregnancy as imprinting of *M6P/IGF2R* was lost in the lineage of primates, colugos and tree shrews (156).

### 5.4 Implications for Animal Models

No gene for human placental protein hormones has orthologous genes in mammals other than anthropoid primates. That does not mean that findings in animal models are without merit for understanding the role of placental hormones in human pregnancy. However, hypotheses generated from models need to be verified in clinical studies or further explored in nonhuman primates (81).

#### 5.4.1 Placental Growth Hormones and Lactogens

Placental lactogens and growth hormones arose through duplication of the genes for pituitary prolactin and growth hormone. They act through similar receptors, i.e., prolactin receptor (PRLR) and growth hormone receptor (GHR). The receptor genes have not undergone duplication, although they did evolve rapidly in parallel with *GH* and *PRL* (87, 151). Therefore, it may be supposed that the placental lactogens of ruminants, rodents, and primates act through similar pathways to effect changes in nutrient availability and maturation of the mammary glands. On the other hand, the most pronounced effects on metabolism and mammary development during human pregnancy are exerted by the placental variant of GH rather than by hPL. Other than anthropoid primates, only sheep and goat have been shown to have a placental growth hormone; it acts locally to promote growth of the uterine glands (117).

The primary function of rodent PLs is to maintain CL function and, acting through the PRL receptor, to promote differentiation of the mammary glands. Although rodent PLs and related proteins have a range of actions, none have such fundamental effects on maternal metabolism as the GH secreted by human placenta. Other products of the gene locus seem not to bind to the PRL receptor but act through non-classical pathways (92). PLs of ruminants stimulate secretion of histotroph from the uterine glands (103). This is important since some substances do not readily cross the epitheliochorial placenta. Uterine gland secretions are rich in uteroferrin, which is taken up by the trophoblast and is an essential source of iron. In addition, ruminant PLs may play a role in mammary gland development. Since it is by no means clear that PLs have evolved to serve the same functions in primates, rodents, and ruminants, extreme care is needed in extrapolating across species.

A further qualifier applies to mouse and rat models. Murid rodents have brief gestations and large litters of poorly developed (altricial) pups. Primates and ruminants have much longer gestations usually with singletons that are well-developed (precocial) at birth (81). Therefore, there is a stretch of several months where placental hormones might regulate maternal physiology in primates and ruminants with no equivalent period in rodents.

#### 5.4.2 Pregnancy-Specific Glycoproteins

PSGs are derived from the carcinoembryonic gene family rather than from pituitary hormones. It is nonetheless remarkable that PSGs have evolved separately in four orders of mammal. Cross-species comparisons should be valuable in determining whether their primary role is to promote trophoblast invasion or to act as viral decoys. Although CEACAMs do act as viral decoys, there is no evidence that PSGS bind to microorganisms (6). The case is stronger for a role in immune tolerance of the invading trophoblast, especially in humans where PSGs, acting through TGFβ1, induce proliferation of regulatory T-cells in the uterus (46, 130). There is growing evidence for a similar mechanism in rodents (140) and there could be a link between PSG expression by the endometrial cups of the horse and the presence of regulatory T-cells in their vicinity (145).

## 6 Conclusions

The placenta expresses a variety of hormones that can affect maternal physiology and fetal development (3). This review has focussed on placental hormones that originated through duplication of existing genes such as those coding for pituitary hormones (**Table 2**). There are several instances of convergent evolution and expansion of gene loci after the initial duplication. Many of the gene products are found at high concentration in maternal blood.

Least contentious in a physiological context are placental hormones responsible for pregnancy recognition and maintenance of the corpus luteum. These include INFT in ruminants, hCG in anthropoid primates, eCG in equids and PLs in murid and cricetid rodents.

There could be a common purpose to the convergent evolution of PSGs in 4 orders of mammal. It is notable that human PSGs and murine Psg23 can activate TGFβ1 and thereby promote proliferation of regulatory T-cells. It has yet to be demonstrated conclusively that the primary function of PSGs is immunosuppression and promotion of trophoblast invasion. However, this is an active area of research (6).

The role of the placental variant of GH in human pregnancy is clear. It reprogrammes maternal metabolism and ensures an adequate supply of glucose to the fetus. A placental GH has been convergently evolved in one family of ruminants where it may play a role in development of the uterine glands.

More perplexing is the significance of placental lactogens. Human PL is secreted at high levels and, though derived from a *GH* gene, shows greater affinity for the PRL than the GH receptor. Yet it seems less important for mammary gland development than pituitary PRL (74). In ruminants, there is some evidence that oPL and bPL affect mammary development, but they play a more important role in uterine gland development and secretion of histotroph. In rodents PLs are mainly important for pregnancy maintenance. An additional complication is the expansion of the PRL gene loci in rodents and ruminants. Many of the gene products seem to act through non-classical pathways (92). Thus, care must be taken in extrapolating work on ruminant and rodent prolactins to human pregnancy.

## Author Contributions

The author confirms being the sole contributor of this work and has approved it for publication.

## Conflict of Interest

The author declares that the research was conducted in the absence of any commercial or financial relationships that could be construed as a potential conflict of interest.

## Publisher’s Note

All claims expressed in this article are solely those of the authors and do not necessarily represent those of their affiliated organizations, or those of the publisher, the editors and the reviewers. Any product that may be evaluated in this article, or claim that may be made by its manufacturer, is not guaranteed or endorsed by the publisher.
